# Highly Tough, Biocompatible, and Magneto-Responsive Fe_3_O_4_/Laponite/PDMAAm Nanocomposite Hydrogels

**DOI:** 10.1038/s41598-019-51555-5

**Published:** 2019-10-21

**Authors:** Jin Hyun Lee, Wen Jiao Han, Hyo Seon Jang, Hyoung Jin Choi

**Affiliations:** 1 Polymer Research Center, Inha University, Incheon, 22212 Republic of Korea; 2 Department of Polymer Science and Engineering, Inha University, Incheon, 22212 Republic of Korea

**Keywords:** Rheology, Gels and hydrogels

## Abstract

Magneto-responsive hydrogels (MRHs) have attracted considerable attention in various applications owing to their smart response to an externally applied magnetic field. However, their practical uses in biomedical fields are limited by their weak mechanical properties and possible toxicity to the human body. In this study, tough, biocompatible, and magneto-responsive nanocomposite hydrogels (MR_NCHs) were developed by the *in-situ* free-radical polymerization of N, N-dimethylacrylamide (DMAAm) and laponite and Fe_3_O_4_ nanoparticles. The effects of the concentrations of DMAAm, water, and laponite and Fe_3_O_4_ nanoparticles in the pre-gel solutions or mixtures on the viscoelastic and mechanical properties of the corresponding hydrogels were examined by performing rheological and tensile tests, through which the mixture composition producing the best MR_NCH system was optimized. The effects were also explained by the possible network structures of the MR_NCHs. Moreover, the morphology, chemical structure, and thermal and mechanical properties of the MR_NCHs were analyzed, while comparing with those of the poly(DMAAm) (PDMAAm) hydrogels and laponite/PDMAAm NCHs. The obtained optimal MR_NCH exhibited noticeable magnetorheological (MR) behavior, excellent mechanical properties, and good biocompatibility. This study demonstrates how to optimize the best Fe_3_O_4_/laponite/PDMAAm MR_NCH system and its potential as a soft actuator for the pharmaceutical and biomedical applications.

## Introduction

Stimuli-responsive hydrogels have attracted considerable attention in various fields, including pharmacy and biotechnology, because of their many advantageous features and the smart property that responds to external stimuli, such as magnetic field, electric field, temperature, pH, and light^1–4^. Hydrogels are materials that possess a three-dimensional polymer network structure, capability of absorbing large quantities of aqueous fluids with swelling and without dissolving in the fluid, biocompatibility, and controllable physical and mechanical properties. Hence, they have been getting popular for many applications, such as biomedicine and soft electronics^5,6^. The smart behaviors of stimuli-responsive hydrogels are caused by the components of the hydrogels, particularly functional micro- or nanoparticles embedded in the composite hydrogels in many cases^7,8^.

Magneto-responsive hydrogels (MRHs) are typically prepared by incorporating magnetic particles into hydrogels. Their morphology and the properties, including deformation and movement, can be regulated remotely via manipulating the amplitude and direction of the applied magnetic field (MF)^9,10^. For these reasons, they are considered potential materials for a range of applications, including drug delivery, regenerative medicine, environmental engineering, and soft actuators and sensors^11–14^. The magnetic particles usually contained in the MRHs are magnetite (Fe_3_O_4_), carbonyl iron (CI), cobalt ferrite, (CoFe_2_O_4_), etc. and their type, size, and content affect the characteristics of the MRHs^14–16^. Magnetite (Fe_3_O_4_) particles used in this study have a superparamagnetic nature, showing no magnetic hysteresis, and they have been used as MRI contrast agents and used in thermal therapy and drug/cell delivery because they have strong magnetic moment and biological inertness and biocompatibility^17–19^.

Despite the superior characteristics of hydrogels, including MRHs, their practical uses in biomedical fields are limited because of the weak mechanical properties and possible toxicity to the human body. Therefore, many studies have focused on the development of tough hydrogels by introducing clay particles, slide-ring structure, double network structure, and so on^20–22^. For the clay particle-incorporated nanocomposite hydrogels (NCHs), the clay particles act as physical crosslinkers with multiple functionalities during the gelation of the particle-dispersed pre-gel mixture, and the hydrogel network is constructed most probably via hydrogen and electrostatic interactions between the polymer chains and clay particles^20,23,24^. Their high mechanical toughness and tensile strength were explained by the large energy dissipation caused by the large-scale deformation of polymer chains attached between the clay platelets. Moreover, some studies have reported that laponite (synthetic clay) nanoparticles, which were adopted in this work, could be degraded into non-toxic products in biological fluids and even help improve cell proliferation and differentiation, which will expand their applicability in biomedical fields^25,26^.

Acrylamide (AAm) or its derivative (i.e., N,N-dimethylacrylamide (DMAAm), and N-isopropylacrylamide (NIPAAm)) based hydrogels have been researched most widely for biomedical applications owing to their excellent hydrophilicity and biocompatibility^27,28^. Recent studies comparing clay-incorporated DMAAm-, NIPAAm-, and AAm-based NCHs reported that DMAAm/clay and NIPAAm/clay NCHs exhibit much higher elasticity and moduli than AAm/clay NCHs due to the stronger interactions between the polymer chains and clay particles^29,30^. Another reason to adopt DMAAm in this work is that DMAAm was reported to show a two times higher lethal dose 50 (LD_50_) and lower toxicity than AAm^31,32^.

This article presents magneto-responsive nanocomposite hydrogels (MR_NCHs) prepared by the *in-situ* free-radical polymerization of N,N-dimethylacrylamide (DMAAm) hydrophilic monomers and two different nanoparticles with different functionality: physically multi-crosslinkable laponite nanoparticles and superparamagnetic Fe_3_O_4_ nanoparticles. The concentrations of all components of the pre-gel mixture for the hydrogels were optimized to obtain the optimal MR_NCH system possessing excellent magneto-responsive performances, mechanical properties, and biocompatibility. The effects of the concentrations on the viscoelastic properties (i.e., dynamic storage and loss moduli) and mechanical properties (i.e., stress and strain at break, Young’s modulus, and toughness) of the corresponding hydrogels were investigated by performing rheological and tensile tests. These effects were also explained by the expected network structures of the hydrogel. Moreover, the morphology, chemical structure, and thermal and mechanical properties of the MR_NCHs were characterized and compared with those of PDMAAm hydrogels and laponite/PDMAAm NCHs. Furthermore, their magneto-responsive performances were investigated by observing their magnetorheological (MR) properties and the responses to magnetic stimulus. As a result, the optimized MR_NCH system was selected, and its biocompatibility was checked using *in-vitro* cytotoxicity tests. This study provides a facile approach to achieve a tough and biocompatible MR_NCH with controllable properties regulated by the network structures affected by the type and amount of the components and by the direction and magnitude of an applied magnetic field. In addition, it is shown that the optimized Fe_3_O_4_/laponite/PDMAAm MR_NCH system has great potential as a soft actuator usable in the pharmaceutical and biomedical fields.

## Results and Discussion

### Preparation of hydrogels

**Figure 1:**
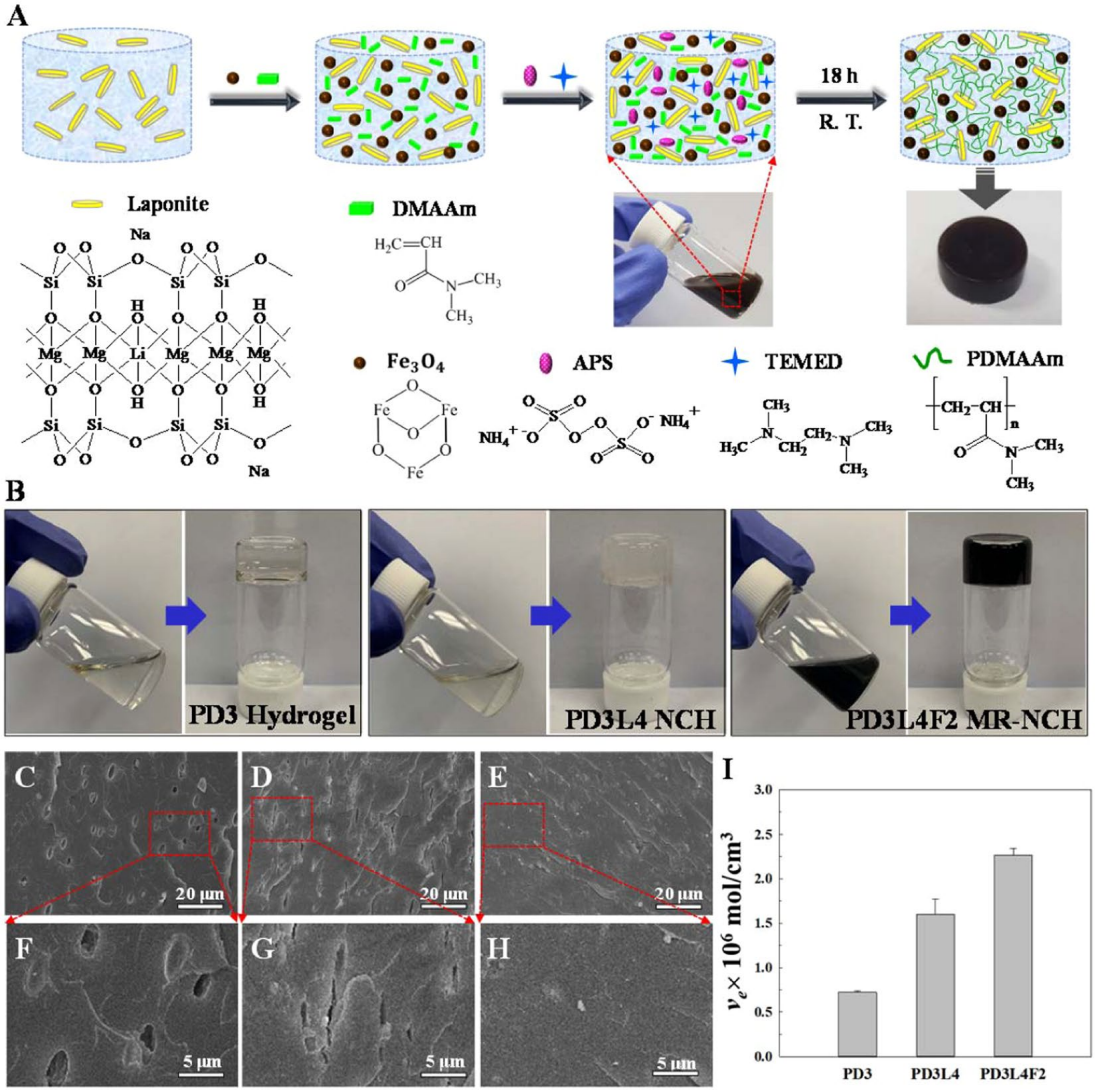
(**A**) Schematic illustration of the preparation procedure of an MR_NCH, and the chemical structures of the components of the pre-gel mixture for MR_NCH. (**B**) Sol-Gel Transition: Photographs taken before and after the gelation of the PD3 hydrogel, PD3L4 NCH, and PD3L4F2 MR_NCH. **(C**–**E)** Cross-sectional SEM images of the freeze-dried PD3 hydrogel, PD3L4 NCH, and PD3L4F2 MR_NCH, respectively and (**F**–**H**) the corresponding magnified images of the part marked by the red square. (**I**) Crosslinking density (*v*_e_) of the PD3 hydrogels, PD3L4 NCHs, and PD3L4F2 MR_NCHs.

Three different types of hydrogels (PDMAAm hydrogels, PDMAAm/laponite NCHs and PDMAAm/laponite/Fe_3_O_4_ MR_NCHs) were fabricated in turn, with optimizing the composition content of the pre-gel solutions or mixtures to obtain the optimal MR_NCH system with the desirable properties. Table 1 lists the compositions of the pre-gel solutions and mixtures used to prepare the three different types of the PDMAAm-based hydrogels along with the names of the corresponding hydrogels. The concentrations of DMAAm monomer, water, and laponite and Fe_3_O_4_ nanoparticles in the pre-gel solutions or mixtures were varied for their optimization needed for the desirable MR_NCHs. Note that the “*” marked PD3L5 NCH and PD3L4F5 MR_NCH means the viscosity of the pre-gel solution or mixture was too high to produce the hydrogels with acceptable quality. The detail synthesis procedure and the name for each hydrogel type are described in the experimental section. Figure 1A presents a schematic diagram of the preparation procedure of an MR_NCH. First, an aqueous solution of laponite nanoparticles was prepared in a vial, and then DMAAm, Fe_3_O_4_, APS, and TEMED were added to the solution and stirred vigorously until the mixture was mixed homogeneously. The black mixture with various components was then polymerized for 18 hrs at room temperature, producing the MR_NCH with the black-brown cylinder shape shown in the photograph. The expected network structure of the resulting MR_NCH is depicted in the illustration. The hydrogel network was formed by attaching the PDMAAm chains to the surface of laponite nanoparticles acting as physical crosslinkers via hydrogen bonds and electrostatic interactions. A similar network formation mechanism could also be expected for the PDMAAm/laponite NCHs and PDMAAm hydrogels, where the hydrogels were synthesized without Fe_3_O_4_ nanoparticles for the NCHs and laponite and Fe_3_O_4_ nanoparticles for the PDMAAm hydrogels.

**Table 1:** Compositions of the pre-gel solutions and mixtures for the three different types of hydrogels.

Hydrogel Name	Hydrogel Type	DMAAm (g)	Laponite (g)	Fe_3_O_4_ (g)	Water (ml)
PD1	H	0.23	0	0	2.07
PD2	H	0.46	0	0	1.84
PD3	H	0.69	0	0	1.61
PD4^*^	H	0.92	0	0	1.38
PD3L1	NCH	0.69	0.03	0	1.90
PD3L2	NCH	0.69	0.06	0	1.90
PD3L3	NCH	0.69	0.10	0	1.90
PD3L4	NCH	0.69	0.13	0	1.90
PD3L5^*^	NCH	0.69	0.16	0	1.90
PD3L4-1^*^	NCH	0.69	0.13	0	1.52
PD3L4-2	NCH	0.69	0.13	0	2.46
PD3L4-3	NCH	0.69	0.13	0	3.20
PD3L4F1	MR_NCH	0.69	0.13	0.035	2.00
PD3L4F2	MR_NCH	0.69	0.13	0.07	2.10
PD3L4F3	MR_NCH	0.69	0.13	0.14	2.24
PD3L4F4	MR_NCH	0.69	0.13	0.21	2.40
PD3L4F5^*^	MR_NCH	0.69	0.13	0.28	2.56

- APS: 0.021 g and TEMED: 30 μL were used for all hydrogels,

- PD: poly(N,N-dimethylacrylamide), L: laponite, and F: Fe_3_O_4_,

- H: hydrogel, NCH: nanocomposite hydrogel, and MR_NCH: magnetic responsive nanocomposite hydrogel,

- *Too high viscosity of the pre-gel solution or mixture to fabricate hydrogels.

Figure 1B shows the appearances before and after the gelation of the PD3 hydrogel, PD3L4 NCH, and PD3L4F2 MR_NCH. It is seen that prior to gelation, the sol states of the pre-gel solutions or mixture exhibit fluidity, while the gel states lose fluidity after gelation. The pre-gel solutions for the PD3 hydrogel and PD3L4 NCH are transparent, while the pre-gel mixture of the PD3L4F2 MR_NCH is opaque black. In addition, the PD3 hydrogel shown to be transparent was sticky and the PD3L4 NCH shown to be slightly milky was quite elastic (rubber-like). In contrast, the MR_NCH shown to be still opaque black was rubber-like and quite tough. Their viscoelasticity is discussed below with their rheological properties. Note that the PDMAAm hydrogels with high adhesion and stickiness were prepared without any crosslinker. The gel formation of the PDMAAm hydrogels was checked many times by carrying out the post-treatments including Soxhlet extraction for 1 week at 100 °C and washing several times with water for 1 month. Despite such severe post-treatments, they retained the gel structure. The PD3 hydrogel survived is presented in Fig. S1 (Supplementary Information).

### Morphology and chemical structure

Figure 1C–E show the cross-sectional scanning electron microscopy (SEM) images of the three different types of freeze-dried hydrogels and Fig. 1F–H present their corresponding higher magnification images: PD3 hydrogel (Fig. 1C,F), PD3L4 NCH (Fig. 1D,G), and PD3L4F2 MR_NCH (Fig. 1E,H). The PD3 hydrogel exhibits some micron-sized and oval-shaped pores, the PD3L4 NCH shows squashed oval-shaped pores, and no pores are shown in the PD3L4F2 MR_NCH. These are somewhat different from the very porous foam-like morphology seen in conventional hydrogels, which is most probably because the three different hydrogels as prepared have a relatively larger content of the PDMAAm (24~30 wt. %) and a smaller water content (70 wt. %). Although the PD3 hydrogel shows a relatively smooth texture and some oval-shaped pores (Fig. 1C,F), the PD3L4 NCH containing laponite nanoparticles (Fig. 1D,G) exhibits a slightly coarse and pleated texture with some squashed oval-shaped pores. The texture is believed to be due to the addition of laponite nanoparticles with a plate-like morphology^33^. In addition, the laponite particles probably squeeze the oval-shaped pores while filling up the voids via multiple crosslinking with PDMAAm chains. Besides, the PD3L4F2 MR_NCH prepared with adding Fe_3_O_4_ and laponite nanoparticles together (Fig. 1E,H) does not exhibit any pores, which is probably because Fe_3_O_4_ nanoparticles with a cube-like or spherical shape^34^ with a size of 50 nm–200 nm (Fig. S2 in the Supplementary Information) fill up even the voids squeezed by laponite nanoparticles. The pleated texture is also seen in the PD3L4F2 MR_NCH, which is probably due to the plate-like morphology of laponite nanoparticles as well. Moreover, no significant aggregation and clustering of the Fe_3_O_4_ nanoparticles are observed in the SEM image. Therefore, it is considered that the Fe_3_O_4_ particles were well dispersed in the MR_NCH.

Figure 1I shows how the addition of laponite and Fe_3_O_4_ nanoparticles affects the crosslinking density (*v*_e_) of the three different types of hydrogels. Their *v*_e_ values were calculated based on their volume equilibrium swelling ratio (*Q*_e_) values (Fig. S3 in the Supplementary Information). As expected, the PD3 hydrogels exhibit the lowest *v*_e_ and the highest *Q*_e_ because they were prepared without laponite nanoparticles acting as crosslinkers. In addition, the *v*_e_ and *Q*_e_ increase and decrease respectively, with adding the laponite particles, which results in the PD3L4 NCHs with the higher *v*_e_ and lower *Q*_e_ compared to the PD3 hydrogels. Besides, the PD3L4F2 MR_NCHs exhibit the highest *v*_e_ and the lowest *Q*_e_; with adding the Fe_3_O_4_ nanoparticles, the decrease in the *Q*_e_ is not significant, although the increase in the *v*_e_ is apparent. This means that the increase in the *Q*_e_ is affected by adding laponite nanoparticles more than adding Fe_3_O_4_ nanoparticle. Moreover, Fe_3_O_4_ nanoparticles are anticipated to be inserted into the void spaces generated by physical crosslinks formed via intermolecular interactions between the PDMAAm chains and laponite nanoparticles, based on the consideration of the mesh size (ξ) depending on the molecular weight between crosslinks (*M*_c_) determined from the *v*_e_. Because the inserted Fe_3_O_4_ nanoparticles most probably occupy a certain amount of the space that water was occupied before adding the nanoparticles, the *Q*_e_ only slightly decreases, however, the *v*_e_ relatively proportionally increases.

**Figure 2:**
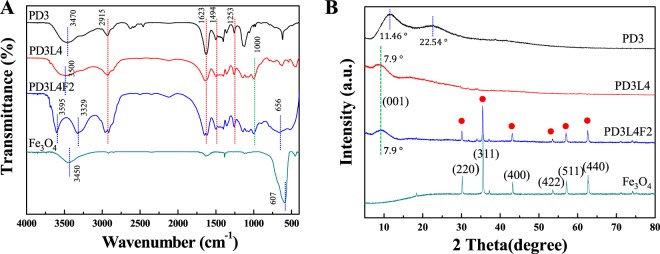
(**A**) FT-IR spectra and (**B**) XRD patterns of the freeze-dried PD3 hydrogel, PD3L4 NCH, PD3L4F2 MR_NCH, and pure Fe_3_O_4_ nanoparticles.

The chemical structure and interactions of three different types of hydrogels were investigated by Fourier-transform infrared (FT-IR) spectroscopy of the freeze-dried hydrogels. Figure 2A shows the FT-IR spectra of the PD3 hydrogel, PD3L4 NCH, PD3L4F2 MR_NCH, and pure Fe_3_O_4_ nanoparticles. All the spectra show the characteristic peaks at 1623 cm^−1^ and 1494 cm^−1^, which are typically observed in PDMAAm included in all hydrogels, attributing to the C=O stretching vibration of the amide I group and to the combination of the N-H bending and the C-N stretching vibrations of the amide II group, respectively^35^. In addition, the peaks at 2915 cm^−1^ and 1253 cm^−1^ in all the hydrogels correspond to the C-H stretching and C-H twisting vibrations of the PDMAAm chains, respectively. Moreover, the Si-O stretching vibration peak of laponite at 1000 cm^−1^ is observed in the spectrum of the PD3L4 NCH, as expected^36^. The shift of the O-H stretching vibration from 3470 cm^−1^ for the PD3 hydrogel to 3500 cm^−1^ for the PD3L4 NCH and 3595 cm^−1^ for the PD3L4F2 MR_NCH after adding laponite nanoparticles demonstrates the intermolecular interactions between the laponite nanoparticles and PDMAAm chains^37^. For pure Fe_3_O_4_ nanoparticles, the characteristic peak at 607 cm^−1^ is assigned to the stretching vibration of the Fe–O bond in the Fe_3_O_4_ crystalline lattice, and the peak at 3450 cm^−1^ corresponds to the O-H stretching vibration of water molecules around the metallic particles. In the spectrum of the PD3L4F2 MR_NCH containing Fe_3_O_4_ nanoparticles, the Fe-O stretching and O-H stretching peaks of pure Fe_3_O_4_ shifts to a higher wavenumber of 656 cm^−1^ and to a lower wavenumber of 3329 cm^−1^,respectively, which demonstrates the intramolecular interactions between the PDMAAm chains and Fe_3_O_4_ nanoparticles and between the Fe_3_O_4_ and laponite particles^38,39^.

Figure 2B presents the X-ray diffraction (XRD) patterns of the freeze-dried PD3 hydrogel, PD3L4 NCH, PD3L4F2 MR_NCH, and pure Fe_3_O_4_ nanoparticles. The PD3 hydrogel exhibits obvious broad bands of PDMAAm at 2θ = 11.5° and 2θ = 22.5° and no periodic pattern due low crystallinity^40,41^. The peak at 2θ = 7.90° being attributed to the (001) crystal plane is seen in the patterns of the hydrogels with laponite nanoparticles (PD3L4 NCH and PD3L4F2 MR_NCH). This peak is shifted from the characteristic peak at 2θ = 5.90°, which corresponds to (001) crystal plane and refers to as the basal layer spacing between the plate-shaped laponite nanoparticles^42^ (Fig. S4 in the Supplementary Information**)**. No significant change in the basal spacing between laponite nanoparticles was observed after the addition of Fe_3_O_4_ nanoparticles. This indicates that the interlayer space formed by the PDMAAm chains attached between the laponite nanoparticles is not noticeably obstructed by even adding Fe_3_O_4_ nanoparticles. This also supports the results that with adding Fe_3_O_4_ nanoparticles, the *Q*_e_ of the MR_NCHs did not significantly decrease, however, the *v*_e_ proportionally increased. On the other hand, the XRD bands at 11.5° and 22.5° of the PD3 hydrogel are not found in those of the PD3L4 NCH and PD3L4F2 MR_NCH. This suggests that the amorphous region of the PDMAAm becomes more dispersed in the hydrogels containing laponite nanoparticles, meaning the disturbance of even small order between the PDMAAm chains^41^. The XRD spectrum of the pure Fe_3_O_4_ particles demonstrates the characteristic diffraction peaks at 30.2°, 35.5°, 43.2°, 53.6°, 57.0°, and 62.7° 2θ, corresponding to the (220), (311), (400), (422), (511), and (440) crystal planes, respectively. Similar characteristic peaks (marked with red solid circles) are observed for the PD3L4F2 MR_NCH, indicating the presence of Fe_3_O_4_ nanoparticles^43^. Overall, XRD and FT-IR spectroscopy of the hydrogels confirmed that the NCHs and MR_NCHs were fabricated successfully, and adding either the laponite or Fe_3_O_4_ particles in hydrogels induces the intermolecular interactions which cause an increase of the physical properties such as *v*_e_ and of the mechanical properties such as stress at break (σ_b_) and toughness.

### Thermal property

**Figure 3:**
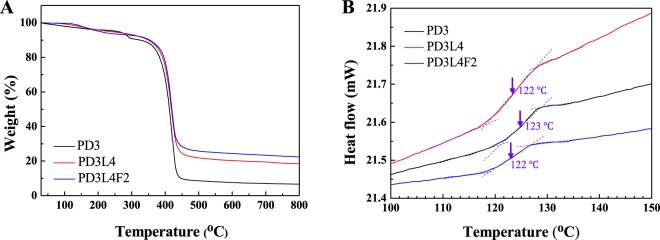
(**A**) TGA and (**B**) DSC thermograms of the freeze-dried PD3 hydrogel (black line), PD3L4 NCH (red line), and PD3L4F2 MR_NCH (blue line).

Figure 3A presents the thermogravimetric analysis (TGA) curves of the freeze-dried PD3 hydrogel, PD3L4 NCH, and PD3L4F2 MR_NCH. The PD3 hydrogel shows a three-stage weight loss pattern in the temperature range from 30 °C to 800 °C, whereas both the PD3L4 NCH and PD3L4F2 MR_NCH exhibit a two-stage pattern. The first stage seen in all curves probably corresponds to the loss of water molecules attached to hydrogels. Only 6~7 wt. % initial weight loss is shown to occur below 280 °C for the three samples. Most of the weight loss occurs from 350 °C to 450 °C, which corresponds to the destruction of the polymer backbone. For the PD3L4 NCH and PD3L4F2 MR_NCH, the decomposition of the polymer backbone began from 360 °C, which is slightly higher than that of the PD3 hydrogel (350 °C). It is thought that the NCH and MR_NCH show slightly improved thermal stability because of not only the network structure established by the interactions between the laponite nanoparticles and PDMAAm chains but also the thermally stable intrinsic nature of the laponite and Fe_3_O_4_ nanoparticles. After 500 °C, all hydrogel samples exhibit the plateau curves and constant values, residual weight percentages. The values of the PD3 hydrogel, PD3L4 NCH, and PD3L4F2 MR_NCH are 6.60 wt. %, 18.4 wt. %, and 21.6 wt. %, respectively. After adding laponite or Fe_3_O_4_ nanoparticles, the thermal stability and residual weight increase. This result supports that the thermal stability of the hydrogels depends on their composition^44,45^.

The DSC thermograms of the freeze-dried PD3 hydrogel, PD3L4 NCH, and PD3L4F2 MR_NCH are shown in Fig. 3B. Only the thermograms in the range from 100 °C to150 °C are shown to accurately determine the glass transition temperature (T_g_) of the hydrogels. The T_g_ values of both the PD3L4 NCH (123 °C) and PD3L4F2 MR_NCH (122 °C) are similar to that of the PD3 hydrogel (122 °C) with no significant difference, regardless of the addition of nanoparticles. These also agree with the reported T_g_ values of other polymer-based composite hydrogels containing laponite nanoparticles^20^. This result demonstrates that the mobility of the PDMAAm chains in the hydrogel network is not significantly affected by the addition of nanoparticles, and the flexible PDMAAm chains crosslinked are distributed uniformly with a long-range scale and maintain the mobility due to the physical crosslinks although a small order between the PDMAAm chains is disturbed.

### Optimization of the concentration of hydrogel components: viscoelastic property

The three different hydrogels by varying the concentration of the hydrogel components (PDMAAm, water, and laponite and Fe_3_O_4_ nanoparticles) were synthesized to optimize their composition for obtaining the hydrogels with desirable properties. First, the optimization was performed based on the viscoelastic properties among the needed properties of MR_NCHs.

Their dynamic viscoelastic properties, characterized by dynamic storage modulus (G’) and loss modulus (G”), were investigated for various concentrations (wt. %) of each component by rheological measurements: strain amplitude sweep and angular frequency sweep tests. Their G’ and G” measured under varying strains ranging from 0.001% to 10% at a constant frequency of 1 Hz in the oscillatory strain amplitude sweep tests are as shown in Fig. S5 (Supplementary Information). Overall, the G’ values that generally denote the stored energy and the elastic properties of materials are higher than those of G” representing the energy dissipation and viscosity property of materials, which is typical behavior of gels. It is also shown that both the G’ and G” maintain plateau values over the entire range of strain. Such a region is the linear-viscoelastic (LVE) region, within which the dynamic moduli are independent of the strain. From the results of the strain amplitude sweep tests, the strain of 1% (the LVE region) was selected for angular frequency sweep measurements.

**Figure 4:**
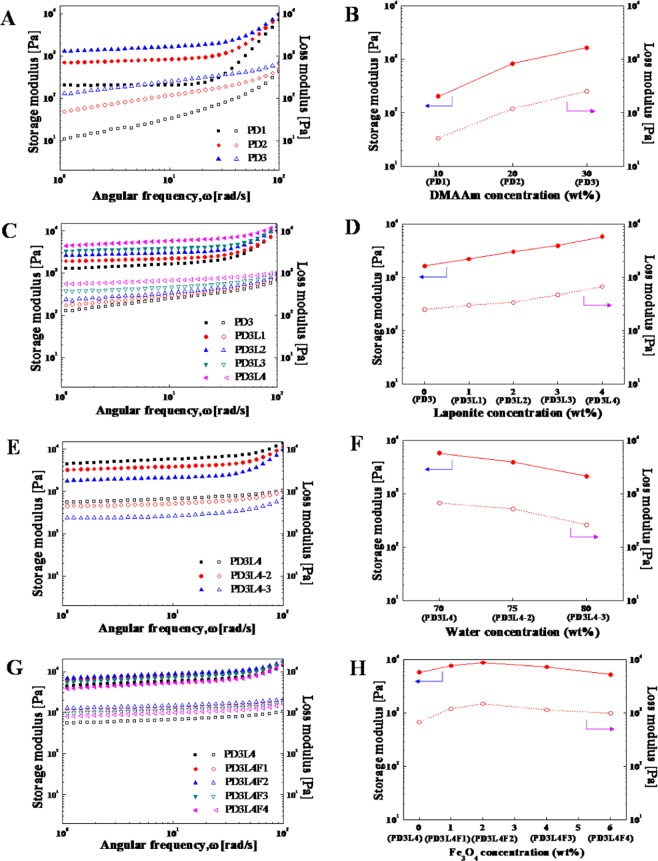
Effect of the concentration of DMAAm (**A**,**B**), laponite nanoparticles (**C**,**D**), water (**E**,**F**) and Fe_3_O_4_ nanoparticles (**G**,**H**) in the pre-gel solutions or mixtures on the storage modulus G’ (closed symbols) and loss modulus G” (opened symbols) of the three different corresponding hydrogels: PD hydrogels, NCHs, and MR_NCHs. Frequency Sweep curves of the hydrogels were obtained at a constant strain of 1% determined from the LVE regions. The G’ and G” taken at the angular frequency of 10.5 rad/s in (**A**,**C**,**E**,**G**) are presented in (**B**,**D**,**F**,**H**).

Figure 4A,C,E,G show the dynamic moduli G’ and G” of the PD hydrogels, NCHs, and MR_NCHs, prepared with different concentrations of components of the corresponding pre-gel solutions or mixtures, as a function of angular frequency. Figure 4B,D,F,H also present the G’ and G” at the selected angular frequency of 10.5 rad/s as a function of the concentration of each component: PDMAAm, water, and laponite and Fe_3_O_4_ nanoparticles, respectively. Note that the hydrogel name corresponding to each concentration is denoted below the concentration in the figures. The G’ and G” are seen to be dependent on the frequency. Overall, the G’ values are higher than the G” values through the frequency range from 1 to 200 rad/s, like the amplitude sweep curves.

Figure 4A,B show that the G’ of the PD hydrogels increases from 0.20 kPa to 1.65 kPa with increasing a DMAAm concentration in the pre-gel solution up to 30 wt. %. This increased G’ is probably due to the denser network structure of PDMAAm hydrogels and lower water content. However, the continued increase in a DMAAm concentration above 30 wt. % causes too high viscosity of the solution, which produces the hydrogels with poor quality or can not provide any hydrogel. Eventually, a DMAAm concentration of 30 wt. % was selected and used for the next optimization steps.

Figure 4C presents the G’ and G” of the NCHs prepared by varying a laponite nanoparticle concentration at the optimized DMAAm concentration as a function of frequency. In addition, Fig. 4D shows these G’ and G” as a function of laponite nanoparticle concentration. Both the G’ and G” increase linearly with increasing a laponite nanoparticle concentration up to 4 wt. %, which also confirms that laponite nanoparticles act as effective crosslinkers to form the networks of the NCHs. When a laponite nanoparticle concentration was higher than 4 wt. %, the pre-gel solution was too viscous to prepare acceptable quality NCHs. As a result, the laponite nanoparticle concentration of 4 wt. % was chosen for further optimization. The G’ and G” of the PD3L4 NCHs prepared from the pre-gel solution with the laponite nanoparticle concentration of 4 wt. % are 5.83 kPa and 0.68 kPa, respectively, at an angular frequency of 10.5 rad/s.

The effect of the water concentration of the pre-gel solutions on the viscoelasticity of the NCHs is shown in Fig. 4E,F. Both the G’ and G” of the NCHs decrease proportionally with increasing the concentration from 70 wt. % to 80 wt. %. The viscosity of the pre-gel solution with a water concentration of 65 wt. % was too high to fabricate the corresponding hydrogels, probably due to the hindered motion of the component molecules for any reaction. As a result, the water concentration of 70 wt. % that provides the PD3L4 NCHs with the highest G’ and G” was reselected for the next optimization round.

Figure 4G,H show the effect of a Fe_3_O_4_ particle concentration of the pre-gel mixture on the G’ and G” of the corresponding MR_NCHs. The dynamic moduli G’ and G” increase when a Fe_3_O_4_ particle concentration increases up to 2 wt. % (the corresponding hydrogel: PD3L4F2 MR_NCH) and later decrease with additionally increasing a Fe_3_O_4_ nanoparticle concentration. The G’ and G” of the PD3L4F2 MR_NCH, the optimal hydrogel, are 8.85 kPa and 1.49 kPa, respectively, at an angular frequency of 10.5 rad/s. The dynamic moduli of the PD3L4F2MR_NCH increased with increasing a Fe_3_O_4_ nanoparticle concentration can be explained not only by the nature of the particles with a higher modulus but also by the contribution of the electrostatic interactions between the laponite and Fe_3_O_4_ nanoparticles to the denser crosslinking structure. However, it is uncovered from this result that the excessive addition of Fe_3_O_4_ nanoparticles can even break the physical crosslinks between the laponite nanoparticles and PDMAAm chains. Although the optimal MR_NCH system was finally selected based on the determined G’ and G”, and the values were not much higher than those of the other MR_NCHs. Therefore, the mechanical properties and MR properties of the MR_NCHs were continuously investigated in the next step to confirm the optimal composition for the best MR_NCH system.

### Mechanical property

**Figure 5:**
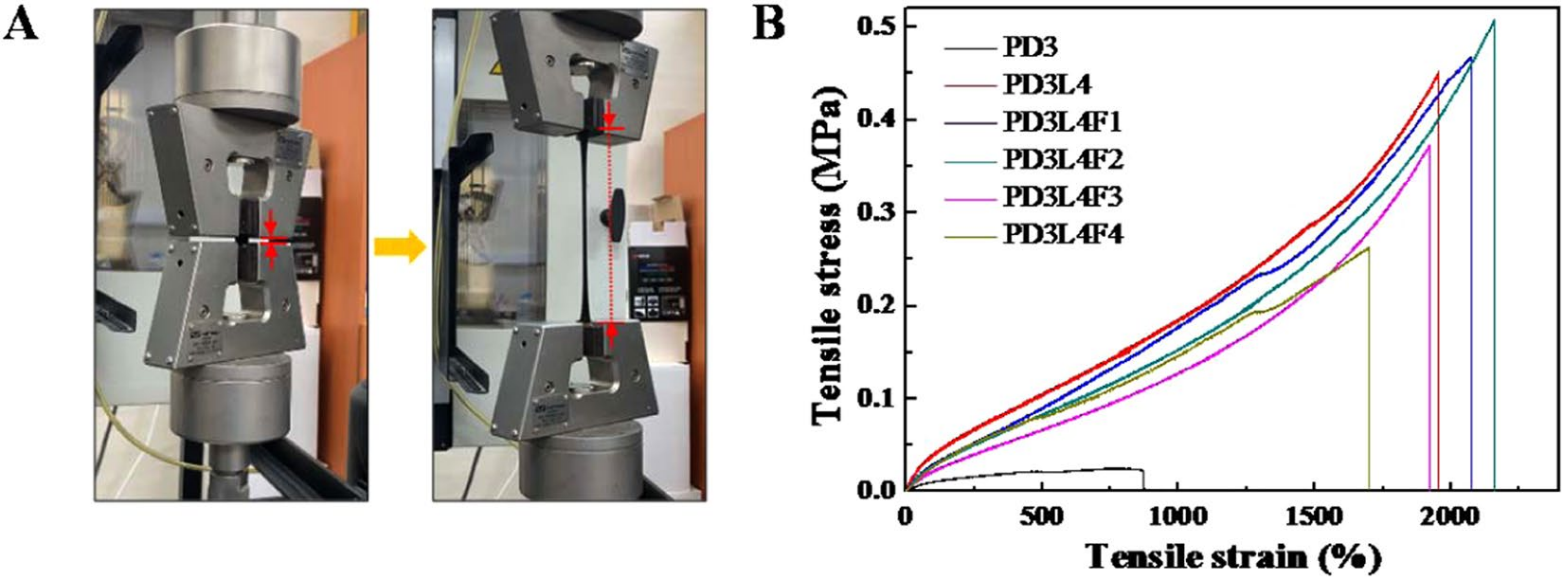
(**A**) Photograph of the elongation of the PD3L4F2 MR_NCH during a tensile test (Left photograph: the initial state of the hydrogel specimen before applying tensile force and right photograph: the fully elongated state of the specimen just before the break). (**B**) Representative stress-strain (SS) curves of the PD3 hydrogel, PD3L4 NCH, and four different MR_NCHs, which were obtained by the tensile tests.

Figure 5A shows the deformation of a prepared hydrogel specimen (here, PD3L4F2 MR_NCH) during a tensile test. The left photograph shows the initial state of the hydrogel specimen before applying the tensile force, and the right photograph shows the fully elongated state of the specimen just before the break. The photographs of the tensile deformation of the three different hydrogel specimens (PD3 hydrogel, PD3L4 NCH, and PD3L4F2 MR_NCH) are presented in Fig. S6 (Supplementary Information). Compared to the NCH and MR_NCH specimens, the PD hydrogel specimen elongates noticeably less until broken. The high extensibility of both the NCH and MR_NCH containing laponite nanoparticles is probably due to the higher crosslinking density formed via the physical interactions between the laponite nanoparticles and PDMAAm chains. The PD3L4F2 MR_NCH is seen to be slightly more elongated than the PD3L4 NCH, which is further discussed below with the quantified experimental data and numerical values of their mechanical properties.

The mechanical properties of the PD3 hydrogels, PD3L4 NCHs, and PD3L4F2 MR_NCHs, which were quantified by tensile tests, are presented as the stress-strain (SS) curves in Fig. 5B and the values are summarized in Table 2. Compared to the PD3 hydrogels, the hydrogels containing laponite nanoparticles (PD3L4 NCHs and PD3L4F2 MR_NCHs) have significantly superior mechanical properties: higher stress at break (σ_b_), strain at break (ε_b_), Young’s modulus (*E*), and toughness. The reason for this result is most probably due to the higher crosslinking density induced by the hydrogen and electrostatic interactions between the PDMAAm chains and laponite particles.

**Table 2:** Mechanical properties of the PD3 hydrogel, PD3L4 NCH, and four different MR_NCHs.

Hydrogel Code	Stress at break (MPa)	Strain at break (%)	Young’s Modulus (MPa)	Toughness (kJ/m^3^)
PD3	0.025	883.4	0.015	162.3
PD3L4	0.445	1986	0.032	3837
PD3L4F1	0.472	2078	0.034	4021
PD3L4F2	0.506	2160	0.035	4198
PD3L4F3	0.353	1925	0.025	2741
PD3L4F4	0.263	1723	0.030	2218

- All values were obtained from five independent tests.

Besides, from the quantitative analysis of the effect of a Fe_3_O_4_ nanoparticle concentration of the pre-gel mixture on the mechanical properties of the MR_NCHs, it was found that there is a critical Fe_3_O_4_ nanoparticle concentration (CFNC) below which the properties decrease and above which the properties decrease. It is speculated that the addition of Fe_3_O_4_ nanoparticles in the hydrogel network can interrupt the physical interactions/crosslinking between the laponite nanoparticles and PDMAAm chains, which decreases the crosslinking density and mechanical strength of the hydrogels (the interruption effect). While, the addition of Fe_3_O_4_ nanoparticles concurrently can mechanically reinforce the hydrogels due to the intrinsic nature of the Fe_3_O_4_ nanoparticles, resulting in higher mechanical properties and crosslinking density (the reinforcing effect). In other words, at a Fe_3_O_4_ nanoparticle concentration below the CFNC (here, 2 wt. %), the reinforcing effect of the Fe_3_O_4_ nanoparticles is more prevalent and the slightly hindered network formation also allows more stress dissipation, which results in the increase in the σ_b_, *E*, ε_b_, and toughness (Fig. 5B and Table 2). On the other hand, at a Fe_3_O_4_ nanoparticle concentration above the CFNC, the more heterogeneous network, generated by the inevitable aggregation of excessively added Fe_3_O_4_ nanoparticles, induces stress defects. In addition, the interruption effect of the excessively added nanoparticles on the formation of the physical network between the laponite nanoparticles and PDMAAm chains is more prevalent beyond the reinforcing nature of Fe_3_O_4_ nanoparticles. Therefore, the σ_b_, *E*, ε_b_, and toughness of the hydrogels decrease with increasing a Fe_3_O_4_ nanoparticle concentration in the nanoparticle concentrations above the CFNC, (Fig. 5B and Table 2). The PD3L4F2 MR_NCHs prepared with the pre-gel mixture showing the CFNC of 2 wt.% exhibit the maximum average values of σ_b_, ε_b_, *E*, and toughness (0.506 MPa, 2160%, 0.035 MPa, and 4198 kJ/m^3^, respectively), indicating that the PD3L4F2 MR_NCHs are the strongest and most stretchable and tough. These two effects of Fe_3_O_4_ nanoparticles can also explain the quantitative analysis results of the viscoelastic properties of the MR_NCHs (Fig. 4H).

### Magnetization, response to magnet, and magnetorheological (MR) property

**Figure 6:**
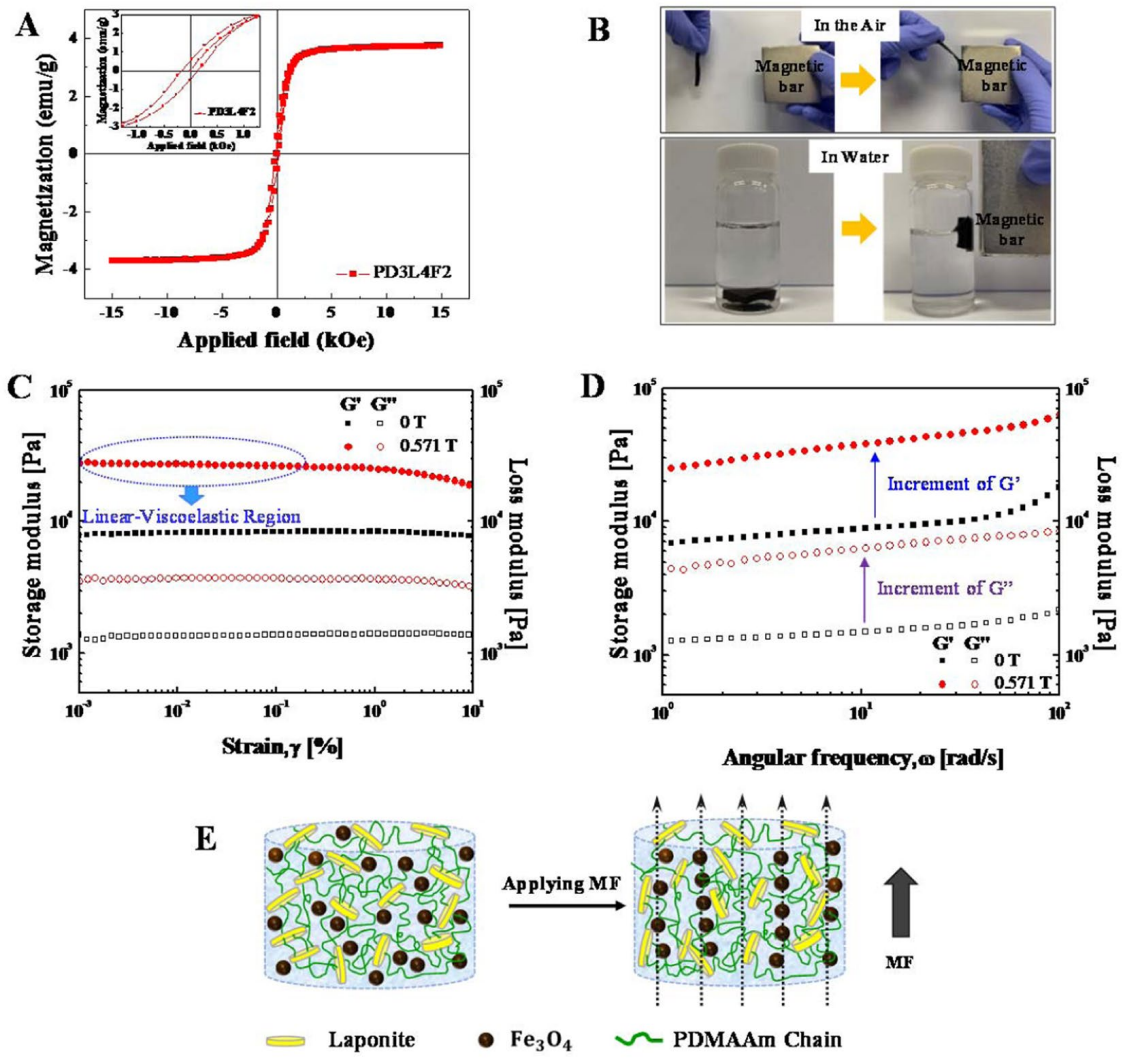
(**A**) Representative magnetic hysteresis loop of the PD3L4F2 MR_NCH in the magnetic field (MF) strength range of −15 kOe to 15 kOe. The hysteresis loop acquired at −1.0 kOe −1.0 kOe is inserted. (**B**) A visual representation of the response of the PD3L4F2 MR_NCH in the air or in water to a magnetic bar. (**C**,**D**) Magnetorheological (MR) properties of the PD3L4F2 MR_NCH: the storage modulus (G’, closed symbol) and loss modulus (G”, opened symbol) as a function, respectively, of strain amplitude and of angular frequency in the absence and presence (0.571 T) of the MF. (**E**) Schematic illustration of the expected network structure of the PD3L4F2 MR_NCH without (left) and with (right) an applied MF. When an MF is applied, the Fe_3_O_4_ nanoparticles in the MR_NCH align along the direction to an applied MF, which also changes the arrangement of laponite nanoparticles and PDMAAm chains attaching to the surface of the laponite nanoparticles.

The addition of Fe_3_O_4_ nanoparticles into the NCHs endows even magnetic responsive properties in addition to the improved mechanical strength and toughness of the hydrogels. First, the magnetization of the optimal MR_NCH system (PD3L4F2) is discussed with its magnetic hysteresis loop. Figure 6A shows the representative magnetic hysteresis loop of the PD3L4F2 MR_NCH, which was measured using a vibrating sample magnetometer (VSM) with an MF strength range of −15 kOe to 15 kOe at 298 K. The inset is the hysteresis loop at −1.0 kOe–1.0 kOe. Although Fe_3_O_4_ nanoparticles are superparamagnetic, the PD3L4F2 MR_NCHs prepared from the pre-gel mixture containing the Fe_3_O_4_ nanoparticles of 2 wt. % exhibit ferromagnetic behavior with hysteresis. However, the area inside the hysteresis loop is quite small as shown in the inset in Fig. 6A, meaning that very low energy is needed to reverse the magnetization. The coercivity and saturation magnetization are 0.15 kOe and 3.7 emu/g, respectively.

Another magnetic property of the PD3L4F2 MR_NCHs was also examined in its response to a magnet. The PD3L4F2 MR_NCH placed in the air or in water was actuated quickly by a magnet (Fig. 6B), demonstrating the potential as a practical device that is controllable with an external magnetic field (MF).

Finally, the magnetorheological (MR) properties of the PD3L4F2 MR_NCHs were examined by measuring the dynamic G’ and G” in the absence and presence of MF, using a rotational rheometer equipped with a magneto-cell producing a homogeneous MF. Figure 6C shows the dynamic moduli G’ and G” of the PD3L4F2 MR_NCHs as a function of strain at a constant frequency of 1 Hz with and without an applied MF. In the absence of an MF, the G’ values are higher than the G” values, maintaining a plateau over the entire strain range, which indicates a well-established network structure of the hydrogels. When a magnetic field of 0.571 T is applied, the Fe_3_O_4_ magnetic nanoparticles most probably tend to form chain-like structures and align along the direction of the applied MF, resulting in a dramatic increase in both the G’ and G” of the MR_NCH. In particular, both the G’ and G” exhibit strain-independent behavior when strain increases up to 0.3%, and they begin to decline slowly after the strain of 0.3%. This nonlinear viscoelasticity behavior is called the Payne effect^46^. From this result, it is thought that the MF-induced network structure of the MR_NCH is altered depending on an applied strain range.

Figure 6D presents the dynamic moduli of the PD3L4F2 MR_NCH as a function of angular frequency, which was determined from the frequency sweep tests performed at a strain of 0.01%. This strain in the LVE region was determined from the amplitude sweep curves. When an MF of 0.571 T is applied to the MR_NCH, both the G’ and G” values increase significantly from the values obtained in the absence of MF, with maintaining a high plateau region over the entire angular frequency range from 1 rad/s to 100 rad/s. This is explained above by the rearrangement of the Fe_3_O_4_ nanoparticles under an MF. In more detail, the Fe_3_O_4_ nanoparticles incorporated in the hydrogel realign along the direction of the applied MF and form a chain-like structure most probably due to the magnetic interactions among the magnetic dipoles developed in the Fe_3_O_4_ nanoparticles. The arrangement of the laponite nanoparticles and the PDMAAm chains attached to the surface of the nanoparticles are simultaneously affected by the alignment of Fe_3_O_4_ nanoparticles, also resulting in their rearrangement and a change in the network structure and then occurring the deformation of the hydrogel network. The expected network structures of the MR_NCH before and after an MF is applied are schematically illustrated in Fig. 6E.

### *In-vitro* cytotoxicity

**Figure 7:**
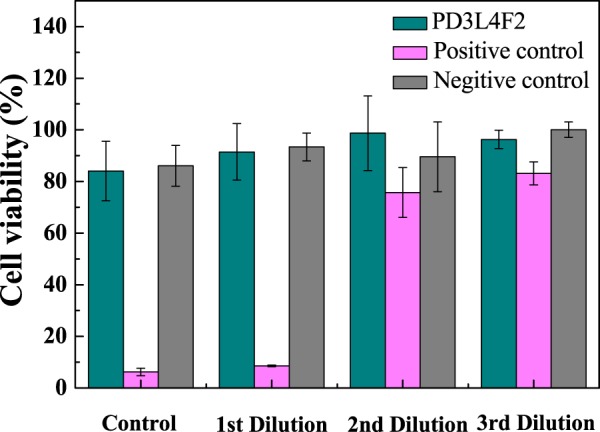
Cell viability of the PD3L4F2 MR_NCH.

The biocompatibility of the optimal MR_NCH system (PD3L4F2) was examined by an *in-vitro* cytotoxicity assay based on the extract dilution tests. Figure 7 presents the viability percentages of NIH/3T3 mouse fibroblasts cells as a function of the extract dilution fractions (1x, 2x, 4x, and 8x) for various extracts of the PD3L4F2 MR_NCHs and the control specimens. In this work, a polyurethane (PU) film and high-density polyethylene (HDPE) film were used as the positive and negative controls, respectively. The positive control specimens exhibit toxicity except for the 8x dilution of the extracts. While the PD3L4F2 MR_NCHs and negative control specimens show cell viability above 80% for the overall dilutions of their extracts, indicating biocompatibility and no toxicity of the PD3L4F2 MR_NCHs.

## Conclusions

This article presents the tough, biocompatible, and magneto-responsive nanocomposite hydrogels (MR_NCHs) that were successfully synthesized by the *in-situ* free-radical polymerization of DMAAm and two different nanoparticles with different functionality: laponite and Fe_3_O_4_ nanoparticles. The concentrations of the pre-gel solution components determining the physical and mechanical properties of the corresponding hydrogels were optimized to obtain the MR_NCHs with the best mechanical and MR properties by examining their rheological, tensile, and magnetorheological properties. The optimal MR_NCH system is the PD3L4F2 MR_NCH exhibiting extraordinary mechanical strength, extensibility, toughness, and MR properties. During the optimization, the hydrogels with better properties were produced by increasing the concentrations of monomer and laponite nanoparticles. When the concentration was too high, however, the viscosity of the pre-gel solutions or mixtures became too high to prepare acceptable quality hydrogels. Besides, it was found that there was a critical Fe_3_O_4_ nanoparticle concentration (CFNC) (here, 2 wt. %), which is probably because the incorporation of Fe_3_O_4_ nanoparticles into the NCHs causes two effects simultaneously. The addition of Fe_3_O_4_ nanoparticles can interrupt the physical crosslinks/interactions between the laponite nanoparticles and PDMAAm chains, decreasing the crosslinking density and mechanical strength (the interruption effect); however, concurrently can reinforce the composite hydrogels due to the intrinsic mechanical nature of Fe_3_O_4_ nanoparticles, resulting in improved mechanical properties (the reinforcing effect). Therefore, at a concentration lower than the CFNC, the reinforcing effect of Fe_3_O_4_ nanoparticles is probably prevalent, but the interruption effect is probably prevalent at a concentration higher than the CFNC. Moreover, when an MF of 0.571 T was applied, the dynamic G’ and G” of the PD3L4F2 MR_NCH was enhanced dramatically, compared to the values in the absence of EF. This can be explained by the strong alignment of Fe_3_O_4_ nanoparticles incorporated in the hydrogel along the direction of the applied MF due to the magnetic interactions between the magnetic dipoles developed in Fe_3_O_4_ nanoparticles. The MF-dependent alignment of Fe_3_O_4_ nanoparticles probably affects the arrangement of the laponite particles and PDMAAm chains, resulting in a change in the network morphology. Based on these results regarding MR-responses of the MR_NCHs, we proposed the expected network structure of MR_NCHs to be formed before and after applying an MF field. Furthermore, the excellent MR properties of PD3L4F2 MR_NCH showed high potential as a practical device controllable with an external MF. Finally, the PD3L4F2 MR_NCH possessing high mechanical and MR properties also exhibited excellent biocompatibility. Therefore, we suggest that the optimal MR_NCH system developed in this study are new candidate materials for soft actuators usable in pharmaceutical or biomedical fields.

## Materials and Methods

### Materials

N,N-dimethylacrylamide (DMAAm, purity ≥ 99.0%), iron oxide (magnetite, Fe_3_O_4_) powders (50–100 nm) and N,N,N′,N′-tetramethylethylenediamine (TEMED, purity ≥ 99.0%) were purchased from Sigma-Aldrich, USA. Laponite XLG (Mg_5.34_Li_0.66_Si_8_O_20_(OH)_4_Na_0.66_) particles were provided by Rockwood Additives Ltd., Cheshire, UK. Ammonium persulfate (APS) was obtained from DEAJUNG, Korea. Deionized water was used as a solvent, and all reagents were used without further purification.

### Preparation of hydrogels

Three different types of hydrogels (PDMAAm hydrogels, NCHs, and MR_NCHs) were prepared by the *in-situ* radical polymerization of DMAAm and laponite and Fe_3_O_4_ particles in an aqueous solution or mixture at room temperature to obtain the optimal MR_NCHs with desirable properties. For the polymerization, DMAAm as a monomer, APS as an initiator, and TEMED as a co-initiator, and laponite nanoparticles as crosslinkers and Fe_3_O_4_ particles as donors of magnetic properties were used. APS/TEMED, a redox system known to promote the formation of APS radicals and decrease the activation energy of polymerization, was used for polymerization at low temperatures^47,48^. The weight ratio of DMAAm, APS, and TEMED was fixed to 1:0.03:0.03 for all the pre-gel solutions and mixtures for three different types of hydrogels. The PDMAAm pre-gel solutions were prepared by adding TEMED and APS to the DMAAm aqueous solution. For the pre-gel solutions of NCHs, laponite nanoparticles as crosslinkers were first dissolved in water and stirred vigorously until a transparent laponite solution was obtained. DMAAm, APS and TEMED were then added sequentially to the solution with continuously stirring. The pre-gel mixtures of MR_NCHs were also prepared using a similar procedure to those of NCHs. The difference is only that Fe_3_O_4_ nanoparticles were added to the aqueous DMAAm-laponite solution, followed by vigorous stirring and sonication to allow the Fe_3_O_4_ nanoparticles to be dispersed uniformly in the mixture. Finally, all the prepared pre-gel solutions or mixtures were left to stand for 18 hrs to be polymerized completely. Table 1 lists the compositions of pre-gel solutions or mixtures for the three different types of hydrogels (PD hydrogels, NCHs, and MR_NCHs). Each hydrogel was named using the first letter of a component contained the hydrogel. The PD, L and F letters in the hydrogel names correspond to PDMAAm, laponite nanoparticles, and Fe_3_O_4_ nanoparticles, respectively. The compositions of the pre-gel solutions or mixtures were optimized by determining the viscoelastic and mechanical properties of the corresponding hydrogel samples. The optimal PDMAAm hydrogels, PDMAAm/laponite NCH, and PDMAAm/laponite/Fe_3_O_4_ MR_NCH were PD3, PD3L4, PD3L4F2, respectively. The physical, mechanical, and MR properties and biocompatibility of the optimal MR_NCH system (PD3L4F2) were characterized to check its potential as a soft actuator material.

### Morphology and chemical structure analysis

The microstructural morphology of the three different types of hydrogels was examined by high-resolution scanning electron microscopy (HR-SEM) (SU-8010, Hitachi, Japan). The freeze-dried hydrogel specimens were sectioned to expose the cross-section to observe their internal structure. In addition, the composition, chemical structure, and possible interactions of the hydrogels were characterized by Fourier-transform infrared spectroscopy (FT-IR, VERTEX 80 V, Bruker) over the wavenumber range from 500 cm^−1^ to 4,000 cm^−1^ and by X-ray diffraction (XRD, DMAX-2500, Rigaku) using Cu-Kα radiation between 5° and 80° 2θ. The morphology and size of the Fe_3_O_4_ nanoparticles were analyzed by transmission electron microscopy (TEM) (CM200, Philips, Netherlands). Moreover, the crosslinking density (*v*_e_) of the hydrogels was calculated by the relationship^49^: *G* = *v*_e_*R*T*Q*_e_^1/3^, where *G* is the shear modulus of the fully swollen hydrogels (3 G = *E*, assuming the Poisson ratio of the hydrogels is 0.5), and *R* and T are molar gas constant and absolute temperature, respectively. *Q*_e_ is the volume equilibrium swelling ratio of the hydrogels [*Q*_e_ = 1 + *ρ*_p_ (*Q*_e_(m) − 1), *ρ*_p_ is polymer density, and *Q*_e_(m) is the mass equilibrium swelling ratio of the hydrogels]. The *Q*_e_(m) of the hydrogels is calculated from the ratio of the weight of the fully swollen state to the weight of fully dried state, based on their swelling measurements.

### Thermal analysis

Thermogravimetric analysis (TGA, TG209F3, Germany) and differential scanning calorimetry (DSC, PerkinElmer 7, USA) were employed for thermal analysis of the hydrogels. TGA was carried out at a heating rate of 10 °C min^−1^ from 20 °C to 800 °C in a nitrogen flow. DSC was also carried out at a heating rate of 10 °C min^−1^ and in a nitrogen flow rate of 10 ml/min. The T_g_ values of the freeze-dried hydrogel samples were obtained from the mid-point of the glass transition in the second heating scan run.

### DC magnetization measurement and magnetic response

The magnetic response of the MR_NCHs was observed visually with a magnet, and their magnetization was measured accurately using a VSM (VSM7307, Lakeshore, USA) at 298 K. The range of the applied magnetic field strengths was from −15 kOe to 15 kOe. The saturation magnetization (the maximum possible magnetization) and the coercivity (the field required to decrease the magnetization to zero after the saturation of magnetization) were determined.

### Rheological and magneto-rheological tests

The rheological properties of the three different types of hydrogels were measured using a rotational rheometer (MCR 302, Anton-Paar, Germany) equipped with 20 mm parallel plates (PP20 geometry) and a magneto-cell (PS-MRD/54, Anton-Paar, Austria), which produces a homogeneous MF. Disc-shaped hydrogel samples (diameter: 20 mm and thickness: 1.2 mm) were prepared for rheological and magnetorheological tests. The strain amplitude and angular frequency sweep tests of the hydrogel samples were performed with and without an applied magnetic field. The angular frequency sweep tests for the samples were carried at a constant strain determined from the LVE regions in the corresponding strain amplitude sweep curve.

### Tensile test

The mechanical properties of the three different types of hydrogels were carried out using a universal testing machine (UTM, Instron 5569, USA) in uniaxial tension at 25 °C and 30% humidity. Cuboid hydrogel specimens (width: 6 mm and thickness: 2 mm) were prepared for the tensile tests. The specimens were clamped vertically with a gap length of 10 mm, and a cross-head speed of tensile force was set to 20 mm/min. The stress-strain (SS) curves of the hydrogel specimens were also obtained from the tensile tests. The stress at break (σ_b_) and strain at break (σ_b_) of the specimens were obtained from the point where the specimen had broken down. The Young’s modulus (*E*) of the specimen was measured from the initial slope of each SS curve. The toughness of the specimen was determined from the area under the SS curve corresponding to the energy required to break the specimen. All the values were obtained from five independent tests.

### *In-vitro* cytotoxicity assay

*In-vitro* cytotoxicity tests of the PD3L4F2 MR_NCH were carried out using the extract dilution method. For comparison, the tests of the positive and negative controls were performed concurrently. Polyurethane (PU) film and high-density polyethylene (HDPE) film were used as a positive control and negative control, respectively. The tests were evaluated by examining the viability of the NIH 3T3 mouse fibroblasts cell line in the extract dilutions of the specimens. Sterilized specimens were placed into a 6-well plate and incubated in cell culture medium for 72 hrs. In parallel, the fibroblasts cell line (1 × 10^4^ cells/100 ul/well) was seeded in 96-well plates and incubated in the culture medium with 10 wt. % fetal bovine serum (FBS) for 24 hrs. The specimen extracts with various dilution fractions (1x, 2x, 4x, and 8x) were fed into the cell and incubated for a further 24 hrs. Finally, the cell viability tests with water-soluble tetrazolium salt (WST, EZ-CYTOX, Dogen) were carried out. The cell viability (%) of all the specimens was determined from the absorbance at 450 nm using a plate reader for various extract dilutions. The cell viability values were obtained from six independent tests.

## Supplementary information

Supplementary Information

## Data Availability

All data acquired or analyzed during this study are included in this manuscript and its Supplementary Information files.
