# Supplementary material for: Highly Tough, Biocompatible, and Magneto-Responsive Fe3O4/Laponite/PDMAAm Nanocomposite Hydrogels
Source: Sci Rep. 2019 Oct 21;9:15024. doi: 10.1038/s41598-019-51555-5 (PMC6803758; doi:10.1038/s41598-019-51555-5)
Supplement: Supplementary file 1 — Supplementary Information [file 41598_2019_51555_MOESM1_ESM.docx]

**Supplementary Information**

**Highly Tough, Biocompatible, and Magneto-Responsive**

**Fe_3_O_4_/Laponite/PDMAAm Nanocomposite Hydrogels**

Jin Hyun Lee^1,§,^*, Wen Jiao Han^2,§^, Hyo Seon Jang^2^ & Hyoung Jin Choi^2,^*

^1^Polymer Research Center, Inha University, Incheon 22212, Republic of Korea,

^2^Department of Polymer Science and Engineering, Inha University, Incheon 22212, Republic of Korea,

*Correspondence and requests for materials should be addressed to J. H. Lee & H. J. Choi (Email: [hannahlee@inha.ac.kr](mailto:hannahlee@inha.ac.kr), [hjchoi@inha.ac.kr](mailto:hjchoi@inha.ac.kr) )

§These authors contributed equally to this work.

**Captions of Supplementary Figures**

**Figure S1**. Photograph of the PD3 hydrogel survived after the post-treatment (Soxhlet extraction for 7 days at 100 ^o^C and washing with water many times for 1 month). No disintegration of the hydrogel is observed.

**Figure S2**. TEM image of Fe_3_O_4_ nanoparticles (the size of 50 nm ~ 200 nm and the cube-like shape).

**Figure S3**. Volume equilibrium swelling ratio of the hydrogels (*Q*_e_) of the PD3 hydrogel, PD3L4 NCH, and PD3L4F2 MR_NCH.

**Figure S4.** XRD pattern of pure laponite nanoparticles.

**Figure S5**. Effect of the concentration of DMAAm (**A**), laponite particle (**B**), water (**C**) and Fe3O4 particle (**D**) in the pre-gel solutions or mixtures on the storage modulus G’ (closed symbols) and loss modulus G’’ (opened symbols) of the corresponding hydrogels: PDMAAm hydrogels, Laponite/PDMAAm NCHs, and Fe_3_O_4_/laponite/PDMAAm MR_NCHs. Strain amplitude sweep tests were performed in the range of strain from 0.001% to 10 % at room temperature.

**Figure S6**. Photographs of the elongation of the PD3 hydrogel (**A**), PD3L4 NCH (**B**), and PD3L4F2 MR_NCH (**C**) in tensile tests. Each left photograph: the initial state of the hydrogel specimen before applying tensile force, and each right photograph: the fully elongated state of the specimen just before the break.

**Figure S1**. Photograph of the PD3 hydrogel survived after the post-treatment (Soxhlet extraction for 7 days at 100 ^o^C and washing with water many times for 1 month). No disintegration of the hydrogel is observed.

**Figure S2**. TEM image of the Fe_3_O_4_ nanoparticles (the size of 50 nm ~ 200 nm).

**Figure S3**. Volume equilibrium swelling ratio (*Q*_e_) of the PD3 hydrogels, PD3L4 NCHs, and PD3L4F2 MR_NCHs.

**Figure S4.** XRD pattern of pure laponite nanoparticles.

**Figure S5**. Effect of the concentration of DMAAm (**A**), laponite particle (**B**), water (**C**) and Fe_3_O_4_ particle (**D**) in the pre-gel solutions or mixtures on the storage modulus G’ (closed symbols) and loss modulus G’’ (opened symbols) of the corresponding hydrogels: PDMAAm hydrogels, Laponite/PDMAAm NCHs, and Fe_3_O_4_/laponite/PDMAAm MR_NCHs. Strain amplitude sweep tests were performed in the range of strain from 0.001% to 10 % at room temperature.

**Figure S6**. Photographs of the elongation of the PD3 hydrogel (**A**), PD3L4 NCH (**B**), and PD3L4F2 MR_NCH (**C**) in tensile tests. Each left photograph: the initial state of the hydrogel specimen before applying tensile force, and each right photograph: the fully elongated state of the specimen just before the break.
